# Rejuvenation of the aged neuromuscular junction by exercise

**DOI:** 10.15698/cst2018.02.123

**Published:** 2018-02-01

**Authors:** Tabita Kreko-Pierce, Benjamin A. Eaton

**Affiliations:** 1 Department of Cellular and Integrative Physiology, University of Texas Health Sciences Center at San Antonio, San Antonio, Texas, USA.; 2 Barshoph Institute of Longevity and Aging Studies, University of Texas Health Sciences Center at San Antonio, San Antonio, Texas, USA.

**Keywords:** neurotrophin, neuromuscular junction, aging, retrograde signaling, exercise

## Abstract

Age-dependent declines in muscle function are observed across species. The loss of mobility resulting from the decline in muscle function represents an important health issue and a key determinant of quality of life for the elderly. It is believed that changes in the structure and function of the neuromuscular junction are important contributors to the observed declines in motor function with increased age. Numerous studies indicate that the aging muscle is an important contributor to the deterioration of the neuromuscular junction but the cellular and molecular mechanisms driving the degeneration of the synapse remain incompletely described. Importantly, growing data from both animal models and humans indicate that exercise can rejuvenate the neuromuscular junction and improve motor function. In this review we will focus on the role of muscle-derived neurotrophin signaling in the rejuvenation of the aged neuromuscular junction in response to exercise.

## INTRODUCTION

Research over the past two decades has uncovered novel roles of skeletal muscle beyond its contractile function. Today skeletal muscle is recognized as a major endocrine organ with the capacity to secrete signals and act on distal targets such as adipose tissue, liver, pancreas, brain and endothelium. In response to environmental or dietary challenges, as well as to organelle and metabolic dysfunctions, the skeletal muscle secretes signals in the form of myokines, myometabolites, neurotrophins and other muscle-derived signals to help maintain the metabolic and physiological homeostasis of the organism 1,2. In addition to communication with remote tissues, it is well established that proper development of the neuromuscular junction (NMJ) requires communication between the muscle and motor neuron. Numerous studies have identified retrograde signals originating from the muscle that are critical for normal synaptic structure and function of the NMJ during development and in the adult 3,4,5,6. Age-dependent declines in motor activity have been documented across species and in humans to represent a significant health issue for the elderly. The contributions of changes in synaptic function at the NMJ to the declines in motor function observed during aging are becoming more appreciated, but the important mechanisms underlying the effects of age on NMJ structure and function remain poorly described. For example, it is unclear the role that retrograde signaling at the NMJ plays in the declines in motor function observed during aging. Muscle-derived signaling molecules, such as neurotrophins and morphogens, are established retrograde signals with powerful effects on NMJ structure and function and, thus, represent candidate signaling systems for the maintenance of motor function during aging. Importantly, exercise induces the muscle expression of many of these neurotrophins providing a molecular explanation for the rejuvenation of the structure and function of the aged NMJ in response to exercise.

## RETROGRADE TROPHIC SIGNALING AT THE NMJ

During development the increase in muscle size due to fiber growth results in a change in the resistance of the growing muscle fiber to the depolarizing input of the motor neuron. This change in the electrical properties requires a precise concomitant change in neurotransmitter release from the presynaptic nerve terminal to maintain faithful depolarization of the muscle. The mechanism coupling muscle growth and synaptic input is the production of muscle-derived retrograde trophic signaling that supports the growth of the synaptic innervation. A beautiful example of this coupling is observed during the development of the *Drosophila* larval NMJ. During the initial ~100 hours of larval development, the surface area of the muscle increases nearly 100-fold, which results in a large reduction in the electrical resistance of the muscle making the muscle harder to depolarize. Despite this change in the muscle, the depolarization of the muscle by the motor neuron is precisely maintained throughout development to insure normal larval motility. To maintain the consistent depolarization of the muscle, the presynaptic nerve terminal releases increasing amounts of neurotransmitter. To facilitate the increase in neurotransmission, the larval NMJ grows by increasing the number of boutons at the synapse 10-fold during developmental growth (Note: Boutons are morphometric structures of the *Drosophila* NMJ that is commonly used as a quantitative measure of synapse size) 7. In these studies, statistical analysis of post-synaptic electrophysiological recordings was utilized to determine precisely the total number of synaptic vesicles utilized to generate the evoked excitatory post-synaptic potential (EPSP) in the muscle. These quantal analyses reveal that the change in neurotransmission was due to the increase in the total number of synaptic vesicles released (i.e. quantal content) and not due to changes in the depolarization of the muscle in response to a single quanta of neurotransmitter (i.e. quantal size), which often indicate changes in post-synaptic neurotransmitter receptor function 8. This terminology has been adopted for this review.

The preciseness of the adjustments in neurotransmitter release during larval growth suggests that the presynaptic nerve terminal is being informed about the increasing size of the muscle. This possibility proposes a signal originating from the muscle that instructs the motor neuron, and the nerve terminal, about the size of the growing muscle. Support for this model was provided by Goodman and colleagues who showed in a series of experiments that muscle-derived bone morphogenic protein (BMP), Glass bottom boat (Gbb) and the presynaptic type II BMP receptor Wishful-thinking (Wit) function in a retrograde genetic pathway required for normal synapse growth; and the lack of either leads to a smaller than normal synapse with reduced neurotransmission 9,10. Although these studies support an important role for retrograde BMP signaling for normal synapse growth during larval development, whether retrograde BMP signaling plays a role in the maintenance of the adult NMJ is not known.

At the mammalian NMJ, a number of neurotrophins (NTs) have been shown to be required for proper development of the NMJ 5,6,11. Interestingly, NTs classically known for their role as promoters of motor neuron survival and maintenance (e.g. BDNF (brain-derived neurotrophic factor), GDNF (glial cell line-derived neurotrophic factor), NT-3 and NT-4) have been found to be expressed in adult skeletal muscles supporting the existence of retrograde trophic signaling at the adult NMJ 12,13,14,15,16,17,18,19. Further evidence that muscle-derived NTs could support the maintenance of adult NMJs comes from several observations. For example, analysis of the NMJs in transgenic mice overexpressing GDNF in muscle revealed hyperinnervation of the muscle 18,20,21. Furthermore, the nerve terminals were larger and more complex in the mice with increased muscle-derived GDNF 21. Interestingly, while overexpression of GDNF results in marked morphological changes of the synaptic terminal, similar overexpression of NT-3 or NT-4 showed no effect in these studies 18. These studies reveal a potent ability of GDNF to drive synapse growth, but its role during the development or maintenance of the NMJ is unclear.

In addition to effects on NMJ morphology, several studies have revealed potent effects of NTs on neurotransmission at the NMJ 6,22,23. *In vitro* and *in vivo* treatment with BDNF, GDNF, NT-3 and NT-4 potentiates both the spontaneous and evoked release of neurotransmitter at the NMJ 24,25,26,27,28. Importantly, these effects of BDNF and NT-4 require presynaptic TrkB receptors supporting that these NTs are functioning as retrograde signals with the ability to modulate neurotransmission 25,29. Mechanistically, GDNF has been shown to potentiate presynaptic release by increasing the expression of frequenin, which in turn increases N-type Ca^2+ ^channel activation leading to enhanced Ca^2+ ^influx into the nerve terminal 27. This effectively links muscle derived NTs to the presynaptic neurotransmitter release machinery. The modulation of Ca^2+ ^influx by muscle-derived retrograde signaling has also been demonstrated at the *Drosophila* larval NMJ in response to genetic or pharmacologic reduction in the sensitivity of the muscle to neurotransmitter, supporting that calcium influx into the presynaptic nerve terminal represents a conserved mechanism of retrograde control of presynaptic neurotransmitter release 3,30,31.

## EXERCISE INCREASES RETROGRADE TROPHIC SIGNALING

One of the most remarkable properties of the NMJ is its ability to maintain normal function in the face of physical stresses such as extended periods of increased physical activity. During increased physical activity, the release of neurotransmitter from the presynaptic nerve terminal must be sufficient for each contraction without exhausting the store of synaptic vesicles. A number of studies have demonstrated that both endurance and resistance training stimulate extensive morphological adaptations of the presynaptic nerve terminal of the NMJ. Examinations of the NMJ morphology of the soleus, extensor digitorum longus (EDL), plantaris and gluteus maximus muscles in both mice and rats revealed that strenuous physical training induces NMJ hypertrophy leading to an increase in the degree and length of nerve terminal branching 32,33,34,35,36. In addition to morphology, exercise training was also found to increase the presynaptic release of neurotransmitter at the NMJs of the EDL and gluteus maximum muscles 34,37. The most likely possibility is that the increase in synapse growth, and the subsequent increase in neurotransmission, is an adaptive response of the NMJ to support the increased demands upon the system.

**Figure 1:**
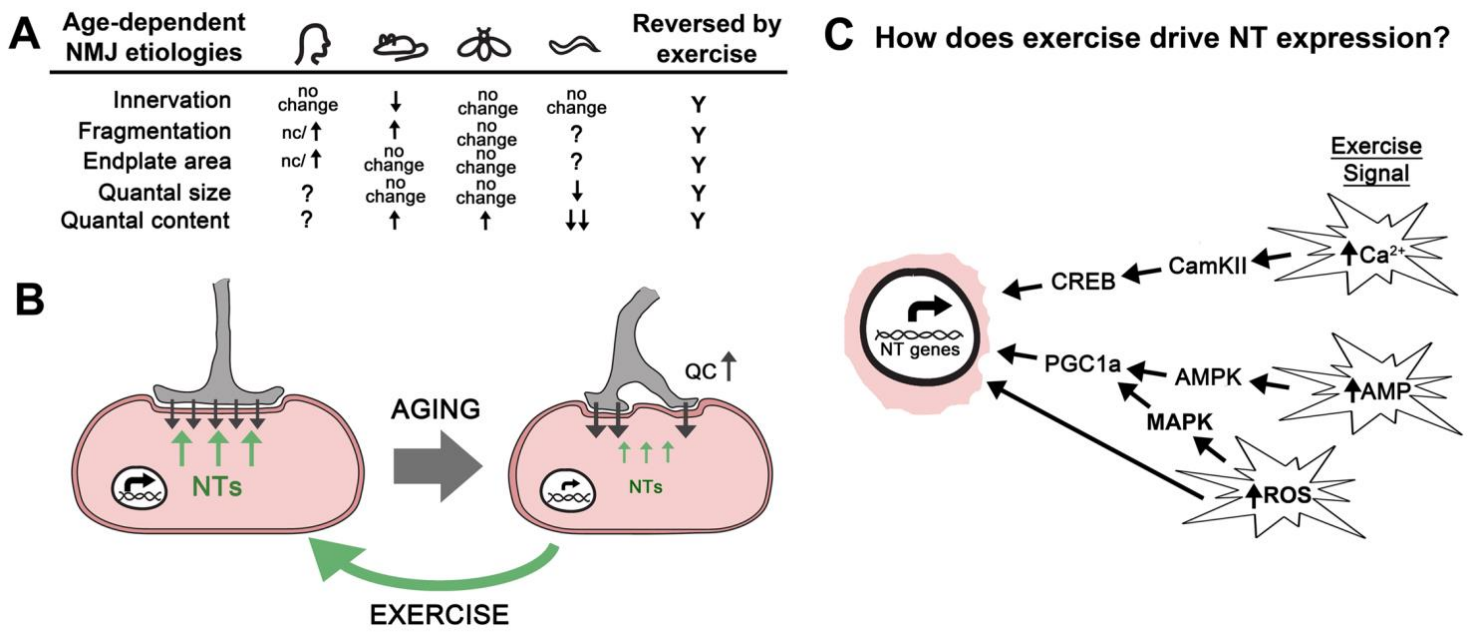
FIGURE 1: Exercise rejuvenates the neuromuscular junction by stimulating retrograde signaling. **(A)** Table contains the changes to the synaptic structure and function of the NMJ during aging from humans, mice, flies, and worms discussed in this review article. Inconsistent results about fragmentation at human NMJs are indicated. Exercise reverses all of these changes. **(B)** Aging results in reduced neurotrophin expression (NT; green arrows) leading to a loss of synaptic morphology and a compensatory increase in neurotransmitter release (quantal content = QC) at the neuromuscular junction (black arrows). Exercise increases the expression of multiple neurotrophin genes (NT genes; green arrows) resulting in improved structure and function of the neuromuscular junction. **(C)** Exercise results in increased cytosolic levels of calcium, reactive oxygen species (ROS), and an increase in the AMP:ATP and ADP:ATP ratios. We propose that these events initiate signaling cascades that result in the expression of neurotrophin (NT) genes via the activity of transcription factors such as CREB and PGC1a. It is expected that mechanical stress and oxidative stress help to modulate these signal transduction pathways. It’s also possible that the oxidation of DNA directly alters expression of NT genes.

The changes observed at the NMJ in response to increased physical activity suggest the existence of an exercise-induced muscle-derived retrograde signal(s) that can modify the morphological and functional properties of the NMJ to adapt to the demands on neurotransmitter release. To that end, several studies have demonstrated elevated BDNF, GDNF, NT-3, and NT-4 levels in skeletal muscles post involuntary and voluntary exercise 38,39,40,41,42,43,44. For example, four weeks of walk-training on a treadmill resulted in an increase in GDNF levels in the skeletal muscle, while hind limb unloaded animals resulted in a decrease in GDNF levels 39. These studies are consistent with the model that NT expression is closely tied to muscle activity, even during bouts of normal activity. In another study, cycling exercise resulted in >10 fold increase of BDNF and GDNF expression in exercised rat soleus muscles 41. Furthermore, low intensity walking preferentially increases GDNF expression in slow twitch soleous myofibers, while the same type of training decreases GDNF expression in the fast-twitch EDL myofibers 42. On the other hand, high intensity exercise caused an increase in GDNF expression in fast-twitch muscle fibers demonstrating that regulation of NT expression in the muscle is a property of both slow- and fast-twitch fibers 44. Importantly, in addition to the changes in skeletal muscle GDNF levels following an increase in physical activity, several studies have demonstrated morphological changes at the same NMJs, demonstrating the relationship between exercise-induced GDNF muscle expression level and exercise-induced NMJ remodeling 43,44. Thus, exercise and increased expression of NTs elicit similar responses at the NMJ both at the level of synaptic morphology and neurotransmission. Collectively, these data support the model that the adaptation of the NMJs to exercise is the result of muscle-derived retrograde NT signaling (**Figure 1B**).

## RETROGRADE SIGNALING DURING AGING

The progressive declines in skeletal muscle mass, referred to as sarcopenia, and muscle strength are broadly observed in mammals and represents one of the first hallmarks of aging. Numerous studies in rodent models have demonstrated that the loss of motor function with age is accompanied with changes in the structural integrity of the NMJ 45, but the contribution of these changes in synaptic morphology to the loss of motor function is still unclear. These morphologies include the increased branching of nerve terminals, the progressive fragmentation of the nerve terminal including the post-synaptic acetylcholine receptor (AChR) clusters, increased extra-junctional AChRs, and myofiber denervation (**Figure 1A**) 46,47,48,49,50,51,52,53,54. Despite the significant fragmenting of the synaptic area, most studies support that total endplate area is unchanged (or even increased) during aging 45,55,56. It should be noted that some of these synaptic changes are due to the re-innervation of new fibers resulting from the continual turnover of myofibers and the loss of fiber type diversity of the motor units 57,58. In humans, studies investigating the morphology of the NMJs formed on the intercostal muscles of the rib cage suggested extensive remodeling of the NMJ, similar to mice 59,60. But a recent study of the NMJs of the lower leg (extensor digitorum, soleus, peroneus longus, and peroneus brevis) from 21 amputees found that these NMJs, when compared to young adult mice, were significantly smaller with thinner pre-terminal axons, had less complex endplates, and showed differential organization of active zone components 61. In addition, these human NMJs were remarkably stable with little fragmentation or morphological change from the 4^th^ to the 10^th^ decade of life 61. This study further investigated the differences in the synaptic proteome between the mouse and human NMJ and found significant differences in the protein composition of the synapses including proteins involved directly in NGF, GDNF receptor, and TRK receptor signaling 61. Interestingly, most of the proteins linked to neurotrophin signaling were found to be less abundant at human NMJs compared to mouse NMJs suggesting that these signaling systems are more operative at the mouse NMJ.

The observation of substantial NMJ remodeling during aging suggested the possibility that neurotransmission might be compromised during aging. But nearly all studies in both mice and flies have reported increased release of neurotransmitter from aged NMJs compared to young NMJs 34,54,56,62,63,64. Perhaps the increase in the release of neurotransmitter from the presynaptic nerve terminal of the NMJ is compensatory for decreasing muscle contraction and/or changes in the sensitivity of the muscle to neurotransmitter. For example, a pharmacological, pathological, or genetic reduction of the postsynaptic neurotransmitter receptor sensitivity at the NMJ leads to a compensatory increase in presynaptic neurotransmitter release (quantal content) across species to insure appropriate muscle depolarization 64,65,66,67,68,69,70,71. These data support the existence of a conserved retrograde signaling system that can alter presynaptic neurotransmitter release. Thus, it is possible that the increase in neurotransmission during aging is the result of impaired homeostatic control mechanisms associated with this form of plasticity. Intriguingly, synaptic homeostasis at the *Drosophila* larval NMJ requires TOR signaling within the muscle, a known cellular signaling system important for adaptive responses to aging 72,73. But most studies investigating the functional properties of the NMJ find that the sensitivity of the muscle to neuotransmitter (i.e. quantal size) is unchanged during aging 34,55,62,64,74,75,76,77 (**Figure 1A**). Thus, the critical signal for the homeostatic increase in neurotransmission is not observed during aging. Furthermore, synaptic homeostasis in *Drosophila* requires the presynaptic Ephexin receptor 65. Analysis of neurotransmission across age in *Drosophila* reveals that mutations in the *ephexin* gene do not block the increase in neurotransmission observed during aging 64. In addition, this study demonstrated that synaptic homeostasis was functioning robustly at older NMJs and that the age-dependent increase in neurotransmission included a re-setting of the homeostatic set-point of the NMJ 64. It is unclear if the increase in neurotransmitter release observed at aged NMJs in rodents also represents a change in the set-point of the synapse.

Another possibility is that the age-dependent increase in neurotransmission observed at the NMJ is in response to declining muscle function. The CM9 muscle group in *Drosophila* is one of 13 muscle groups required for the extension of the adult proboscis during feeding 78. Analysis of the velocity of proboscis extension revealed a steady decrease in velocity across age that correlated well with other measures of locomotion 79. Because the release of neurotransmitter is found to increase at the CM9 NMJ during the same period 64, it is possible that the increase in neurotransmission is compensatory for the reduced muscle function. Kreko-Pierce and colleagues fed adult flies the S107 drug to improve CM9 muscle function during aging to see if this was sufficient to block the increase in neurotransmission 79. Studies from rodents have shown that the age-dependent oxidation of the ryanodine receptor (RyR) results in the dissociation of the FK506-BP2/Calstabin protein from the RyR resulting in impaired excitation:contraction coupling and reduced muscle contraction 80,81,82,83,84. The S107 drug re-establishes the binding of FKBP2-506/Calstabin to the oxidized RyR resulting in a reversal of the age-dependent declines in muscle function 84. Aged flies fed S107 for one week had a complete reversal of the declines in proboscis extension velocities and a reduction neurotransmission 79. In control experiments it was observed that feeding S107 to young flies had no effect on proboscis extension velocities or neurotransmission. These data support the model that the age-dependent increases in neurotransmission observed at the CM9 NMJ is in response to reduced muscle function and suggests the existence of a muscle-derived retrograde signal that normally informs the motor neuron about muscle contraction.

## AGING, EXERCISE, AND NEUROTROPHINS

Given the important role of muscle-derived neurotrophin signaling on NMJ structure and function, it seems possible that changes in muscle-derived neurotrophin signaling during aging are responsible for the changes in synapse function and morphology. Although there is paucity of data surrounding NT expression during aging, there are studies showing that neurotrophin gene expression in muscles declines with age, including *NT-3, NT-4,* and *BDNF* 85,86,87,88,89, although gene encoding *GDNF* increases expression during aging 90,91. Thus it is possible that the change in neurotrophin gene expression in aging muscle contributes to the loss of synapse structure and function of the NMJ during aging. In support are the similar synaptic phenotypes of mice harboring mutations in the *trkB* gene (encoding the receptor for BDNF and NT-4) the NT-4 KO mouse 86,92. Because NT expression is sensitive to muscle activity, this model would predict that exercise should have positive effects on NMJ structure and function. This is consistent with what has been observed in rodent models where exercise has been found to reverse the age-related morphological changes of the NMJ including active zone structures and post-synaptic receptors (**Figure 1B**) 46,93,94. In addition to morphology, there is also evidence that exercise in aged animal leads to a more youthful functional profile 34. Taken together, these data support the model that exercise-dependent increases in NT gene expression in skeletal muscles is a significant contributor to the positive effects of exercise on synapse function and structure at the NMJ.

Currently it is unknown how muscle activity might increase NT gene expression. It is established that increased physical activity activates a number of important signaling systems within the muscle resulting in a change in muscle metabolism and cellular physiology to meet the demands of increased activity 95 (**Figure 1C**). For example, the increase in muscle contraction is predicted to result in an increase in the ratios of AMP:ATP and ADP:ATP leading to the activation of AMP Kinase (AMPK) 96,97. The activation of AMPK is known to increase the transcription of genes predominantly through the activation of the transcriptional co-factor PGC-1α (**Figure 1C**). It is currently unknown if this signaling system is responsible for the increases in NT expression in muscle observed after exercise. Interestingly, PGC-1α has been shown to be required for the increase in expression of the myokine ENDC5, which is cleaved and secreted as irisin from the muscle after exercise 98. In addition to changes in adipose tissues, increased peripheral irisin is able to increase BDNF expression throughout the brain 99. Perhaps increased peripheral irisin levels can also up-regulate BDNF expression in motor neurons providing the benefits of retrograde BDNF signaling via ENDC5.

It is also known that the increase in contraction will lead to an increase in cytosolic Ca^2+^ levels. In the muscle, this increase in calcium is known to activate CamKII leading to the phosphorylation and activation of the CREB transcription factor 100,101. Importantly, CREB binding sites are found in the promoter regions of NT-3, BDNF, and GDNF, although whether this signal transduction pathway is involved in the effects of exercise on NT expression has not been demonstrated 102,103,104,105. Thus, it’s possible that increased actions of the contractile machinery during exercise could lead to an increase in NT expression via the activation of CamKII and subsequent phosphorylation of CREB. In addition, exercise is also known to increase reactive oxygen species (ROS) leading to the activation of ROS-sensitive signaling systems such as MAPK, or by directly altering promote activity 106,107,108. It’s also possible that these signals must coincide in order to drive NT gene expression.

It’s important to consider how aging affects these exercise-related signaling systems and whether this plays a role in the changes in NT expression with age. The effects of oxidative stress on cellular physiology have been extensively reviewed 109, so we will focus our discussion on calcium signaling. Many studies have shown that calcium handling changes during aging in the muscle fiber leading to compromised contraction 110,111,112,113. Importantly, aged muscles have reported both a loss of L-type calcium channel function and impaired sarcoplasmic reticulum discharge during depolarization, which would predict that during contraction peak cytosolic calcium levels are reduced compared to young muscles, possibly leading to altered calcium signaling (i.e. CamKII) 111,112,113,114,115,116,117,118. Other studies suggest that resting cytosolic calcium signaling could be increased due to a calcium leak from the sarcoplasmic reticulum due to oxidation and CamKII phosphorylation of the ryanodine receptor 84,119. What effects these changes in calcium handling have on calcium-dependent signaling processes within the muscle cell, especially CamKII, should be a focus of future research.

Recall that expression of *NT-3, NT-4,* and *BDNF* genes all decline during aging, except for GDNF which has been reported to increase with age 85,86,87,88,89,90,91. This is also clearly different than what occurs during exercise when the expression of these NTs genes changes in unison. But it is interesting to note that GDNF expression, which has strong positive effects on neurotransmission 27, is up-regulated during aging. This suggests that the increase in neurotransmission observed at aged NMJs is the result of increased GDNF retrograde signaling. We would predict a similar scenario at the aging fly NMJ although the identity of the retrograde trophic signal is unknown. It is possible that the altered calcium signaling in the aging muscle is responsible for the increase in GDNF expression, similar to what occurs during exercise. But because the increased calcium with age is not accompanied with other potential signals generated by exercise, such as mechanical stress or an abrupt increase in reactive oxygen species, the response in terms of NT expression is incomplete. Because of the therapeutic potential of increased NT expression, it will be important to identify the important signaling systems involved in the responses of the muscle to exercise. This will include deciphering the signaling mechanisms important for the regulation of NT genes in the muscle in response to exercise. Targeting these mechanisms would be expected to help both the elderly patients struggling with mobility issues and patients suffering from neuromuscular disorders that target the NMJ such as amyotropic lateral sclerosis (ALS) and muscular dystrophy (MD).

## Abbreviations

BDNF: brain-derived neurotrophic factor
BMP: bone morphogenic protein
EDL: extensor digitorum longus
GDNF: glial cell line-derived neurotrophic factor
NMJ: neuromuscular junction
NT: neurotrophin
RyR: ryanodine receptor
